# Endoscopic ultrasonography-guided removal of a migrated stent from the abdominal cavity after transgastric endoscopic ultrasonography-guided drainage of a biliary leak

**DOI:** 10.1055/a-2707-3460

**Published:** 2025-10-16

**Authors:** Tomoya Takahashi, Yusuke Takasaki, Takumi Okuaki, Ippei Ikoma, Yasuhisa Jimbo, Toshio Fujisawa, Hiroyuki Isayama

**Affiliations:** 1 12847 Gastroenterology, Graduate School of Medicine, Juntendo University, Tokyo, Japan; 2 12737 Gastroenterology, Graduate School of Medicine, Chiba University, Chiba, Japan; 3 Division of Gastroenterology, Department of Medicine, Faculty of Medicine, Chulalongkorn University, Bangkok, Thailand

Interventional endoscopic ultrasonography (EUS) is a dynamic and advancing field within endoscopy, but the procedures can have severe complications, such as stent migration, bleeding, perforation, and peritonitis. Migration of a transluminal drainage/anastomotic stent into the abdominal cavity is one of the most severe adverse events of interventional EUS 1 2 . Most cases require surgical management, with few cases of successful removal of the migrated stent having been reported 3 4 . We present a case in which a stent that had migrated into the peritoneal cavity was removed endoscopically under EUS guidance with grasping forceps.

A 76-year-old woman underwent EUS-guided hepaticogastrostomy (EUS-HGS) with a plastic stent for a benign biliary stricture, but biliary leakage was noted the day after the procedure ( Fig. 1 ). Transgastric EUS-guided drainage of the bile leakage was performed with a 7-Fr × 12-cm double-pigtail stent; however, we found that the stent had almost completely migrated into the peritoneal cavity, despite attempted endoscopic removal. We therefore decided to remove the migrated stent from the abdominal cavity through a guiding sheath (Endosheather, PIOLAX, Yokohama, Japan) under fluoroscopic guidance with biopsy forceps, but this also failed. Grasping under fluoroscopic guidance was difficult because it was a two-dimensional view; however, we then detected the migrated stent on an EUS image ( Fig. 2 ), and managed to successfully grasp it with biopsy forceps ( Fig. 3 ) and successfully extract it ( Video 1 ).

**Fig. 1:**
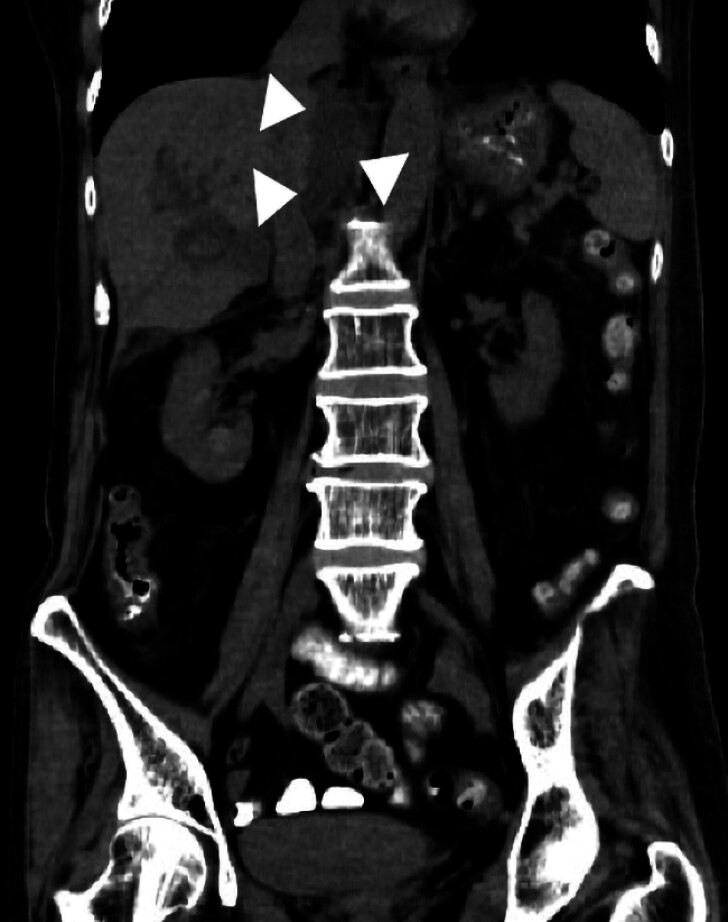
Computed tomography image showing biliary leakage around the liver on day 1 after the endoscopic ultrasonography-guided hepaticogastrostomy procedure.

**Fig. 2:**
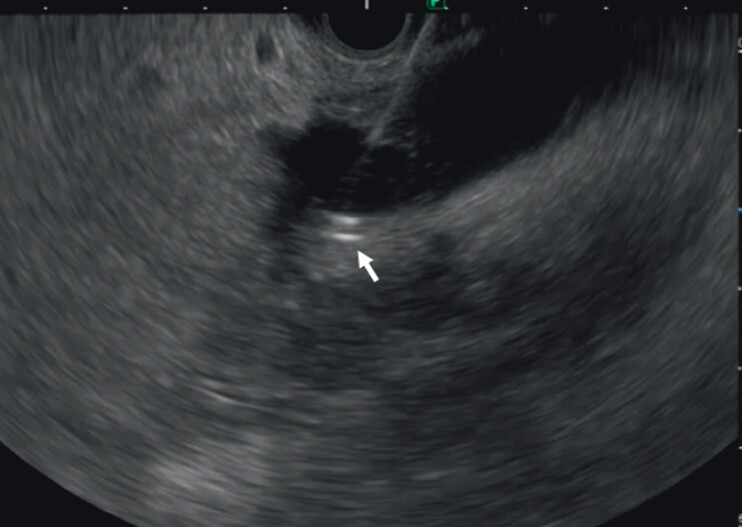
Endoscopic ultrasonography image showing the migrated stent.

**Fig. 3:**
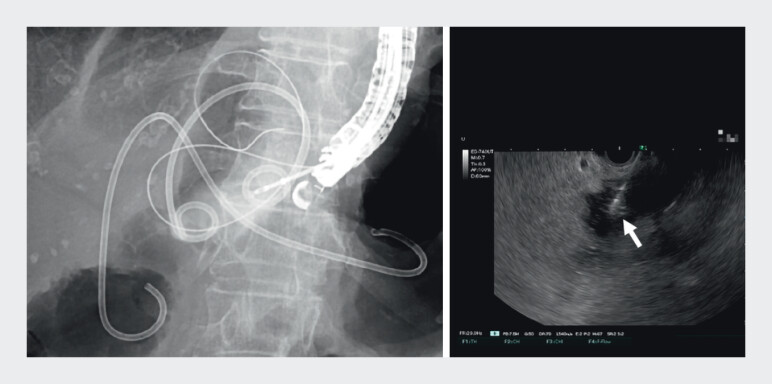
Fluoroscopic and endoscopic ultrasonography (EUS) images showing the migrated stent being grasped under EUS guidance using a sheath (Endosheather, PIOLAX) and biopsy forceps.

A migrated stent placed for endoscopic ultrasonography (EUS)-guided drainage of biliary leakage is grasped and extracted using a sheath and biopsy forceps under EUS guidance.Video 1

Removal of a migrated plastic stent from the peritoneal cavity under EUS guidance has not been previously reported. During removal of migrated stents under fluoroscopic guidance, there are possible risks of grasping other organs, such as the omentum, ligaments, and vessels; however, EUS-guided removal procedures under fluoroscopy avoid these risks. This EUS-guided stent retrieval technique, as reported here, should be considered when a transluminal drainage/anastomotic stent has migrated into the abdominal cavity during an EUS-guided drainage/anastomotic procedure.

Endoscopy_UCTN_Code_CPL_1AL_2AD

## Notes

Endoscopy E-Videos https://eref.thieme.de/e-videos
*E-Videos* is an open access online section of the journal *Endoscopy* , reporting on interesting cases and new techniques in gastroenterological endoscopy. All papers include a high-quality video and are published with a Creative Commons CC-BY license. Endoscopy E-Videos qualify for HINARI discounts and waivers and eligibility is automatically checked during the submission process. We grant 100% waivers to articles whose corresponding authors are based in Group A countries and 50% waivers to those who are based in Group B countries as classified by Research4Life (see: https://www.research4life.org/access/eligibility/ ). This section has its own submission website at https://mc.manuscriptcentral.com/e-videos .
